# A more general role for WR-2721 in cancer therapy.

**DOI:** 10.1038/bjc.1980.150

**Published:** 1980-05

**Authors:** J. M. Yuhas


*Br. J. Cancer* (1980) **41**, 832

***Short Communication***

### A MORE GENERAL ROLE FOR WR-2721 IN CANCER THERAPY

J. M. YUHAS

*From the Cancer Research and Treatment Center and Department of Radiology, University of New Mexico, Albuquerque, New Mexico* 87131, *U.S.A.*

Received 29 October 1979 Accepted 24 January 1980

FOR A NUMBER of years WR-2721 or S-2-(3-aminopropylamino) ethylphosphorothioic acid has been being considered as a potential means of selectively reducing radiation injury to normal tissues, while leaving solid tumours to suffer the full effects of the exposure (Yuhas, 1980*a*). This drug is readily absorbed by most normal tissues (Washburn *et al.*, 1974) and can increase their radiation resistance by factors of up to 3. By contrast solid tumours absorb barely detectable quantities of the drug, and are therefore not radioprotected (Yuhas, 1980*a*). This differential absorption and protection was originally assumed to be the product of differences in normal and tumour tissue vascularity, but more recently it has been shown that normal tissues (with the exception of the CNS) actively concentrate WR-2721 against a gradient, whereas solid tumours passively absorb it or, at most, concentrate it at a far reduced rate (Yuhas, 1980*b*).

Whatever the final resolution of the mechanisms involved, it is now apparent that WR-2721, and other drugs like it, might have a more general application in cancer therapy than we originally envisaged (Yuhas & Storer, 1969). We would like to expand our original proposal (Yuhas & Storer, 1969) to state that WR-2721 should selectively protect normal tissues (except the CNS) against any form of cancer therapy which involves either radiation or alkylating agents or their combination. We have shown recently that injection of WR-2721 30 min before injection of nitrogen mustard ($HN_2$) increases the resistance of mice to $HN_2$-induced mortality by a factor of 2·0, but does not alter the sensitivity of the Line 1 lung carcinoma they bear to $HN_2$-induced growth delay (Yuhas, 1979*a*). The same pattern was observed with another alkylating agent, *cis*-platinum: resistance to *cis*-platinum-induced nephrotoxicity was increased by a factor of 1·7, but none of the 3 solid tumours studied showed altered resistance to *cis*-platinum following WR-2721 pre-treatment (Yuhas & Culo, 1980). Since similar patterns have been obtained with cyclophosphamide (Yuhas, 1980*b*) and L-phenylalanine mustard (Yuhas, unpublished observations) it would appear that WR-2721 could prove to be an effective adjunct to alkylating-agent chemotherapy.

The point of the present argument is not merely whether WR-2721 would be effective in radiotherapy or alkylating-agent chemotherapy, but whether it would be effective in *any* type of therapy involving one and/or the other treatment modality. To test the possibility that WR-2721 could selectively protect normal tissues against the toxic interaction of radiation and alkylating agents, we transplanted the 3M2N mammary carcinoma ($5 \times 10^6$ cells) into the right hind leg of 6 groups of 8 female Fisher 344 rats each $70 \pm 5$ days old, and initiated therapy when their tumours had grown to $\sim 9$ mm, *i.e.* 14 days later. Rats were given an injection of saline (0·75 ml) or WR-2721 (200 mg/kg; Sample AN) in an equivalent volume, followed 30 min later by X-rays (0 or 2500 rad) and/or cis-dichlorodiammineplatinum (Cis-Plat, Bristol Laboratories, Syracuse, New York). The 250kVp X-rays were delivered at a rate of 164 rad/min to the tumour-bearing leg, the remainder of the body being shielded by lead. Immediately after exposure, the rats were given a single i.p. injection of 0 or 5 mg/kg of Cis-Plat (0·005 ml/g body wt). For the next 45 days, the skin reactions were scored 3× weekly, and at the same time the tumours were sized with Vernier calipers. Skin response was expressed as the peak skin reaction during the first 45 days after irradiation (usually Days 20–25), whilst tumour response is expressed as the treatment-induced delay in time required for the tumours to grow 4 mm beyond their size at the time of treatment. As shown in the Table, in the absence of WR-2721 pre-treatment, the combination of radiation (2500 rad) and Cis-Plat (5 mg/kg) shows greater than additive injury to the tumour, but unfortunately the same is observed in the skin. However, if the rats were pre-treated with WR-2721 (200 mg/kg), this toxic interaction is eradicated in the skin, but remains fully expressed in the tumour (Table). Work currently in progress in our laboratory indicates that similar results will be obtained with other more critical normal tissues, such as the kidney.

In the experiment described above, WR-2721 is effective against both arms of the therapy, but this does not have to be the case for this approach to be effective. Even if we assume that WR-2721 will not alter hyperthermic injury, it should prevent synergistic interactions of radiation and hyperthermia in normal tissues, merely by reducing the radiation-injury component. We do not know what effect hyperthermia would have on the distribution and metabolism of WR-2721, but this potential problem could be circumvented by giving WR-2721, radiation, and then hyperthermia, *i.e.* WR-2721 will have performed its function before the heat was applied.

TABLE.—*Effects of WR-2721 pre-treatment (200 mg/kg, i.p.) on the interaction of X-rays (2500 rad) and cis-platinum (5 mg/kg) in producing skin and tumour injury in Fisher 344 rats bearing the 3M2N mammary carcinoma*

| Treatment* | Peak skin injury† | Tumour growth delay (days)‡ |
|---|---|---|
| Cis-Platinum | 0 | 6·1 ± 0·52 |
| Radiation | 2·3 ± 0·31 | 4·1 ± 0·23 |
| Rad. + Cis-Plat. | 4·0 | 15·1 ± 0·97 |
| WR-2721 + Cis-Plat. | 0 | 6·1 ± 0·81 |
| WR-2721 + Rad. | 0 | 5·0 ± 0·77 |
| WR-2721 + Rad. + Cis-Plat. | 0·76 ± 0·15 | 14·2 ± 0·46 |

* WR-2721 (200 mg/kg) given i.p. 30 min before radiation and/or Cis-Plat; Cis-Plat given as an i.p. injection alone or within 5 min after radiation or 30 min after WR-2721.

† 1 = erythema; 2 = dry desquamation; 3 = moist desquamation; and 4 = ulceration.

‡ Delay in time required by treated 3M2N tumours to grow 4 mm beyond the size at the time of treatment.

As a last point, combinations of WR-2721 and non-conventional radiations may prove more effective than either approach alone, because each is able to compensate for the others' deficiencies. If a situation is considered in which both the spinal cord and the tissues immediately adjacent to the tumour are dose-limiting, the effectiveness of WR-2721 in improving the treatment of this tumour would be limited by cord tolerance because WR-2721 does not protect the spinal cord (Yuhas, 1979*b*). Similarly, the effectiveness of pions would be limited by the radiosensitivity of the immediately adjacent normal tissues which would have to be included in the peak treatment volume. The combination of WR-2721 and pions, however, would not suffer excessively from either limitation, because one could reduce damage to the spinal cord by placing it in the plateau region of the pion depth–dose curve and reduce radiosensitivity of the tissues immediately adjacent to the tumour with WR-2721.

In summary, this approach of reducing normal tissue toxicity would appear to have a more general application than we originally proposed (Yuhas & Storer,

1969). Although WR-2721 is well tolerated by patients (Kligerman *et al.*, 1980) and appears to be radioprotective in them (Sugahara & Tanaka, 1980) it should not be considered the final solution to this problem. There still exists much room for drug improvement, both qualitatively and quantitatively.

This research was supported by Grants CA 21074-04 and 1RO1-CA-19326-02 from the National Cancer Institute, DHEW.

### REFERENCES

KLIGERMAN, M. M., SHAW, S., SLAVIK, M. & YUHAS, J. M. (1980) Phase I clinical studies with WR-2721. *Cancer Clin. Trials.* (In press.)

SUGAHARA, T. & TANAKA, Y. (1980) Clinical experiences of chemical radiation protection in tumor radiotherapy in Japan. *Cancer Clin. Trials.* (In press.)

WASHBURN, L. C., HAYES, R. L., CARLTON, J. & YUHAS, J. M. (1974) Distribution of WR-2721 in normal and malignant tissues of mice and rats: Dependence on tumor type, drug dose and species. *Radiat. Res.*, **59**, 475.

YUHAS, J. M. (1979*a*) Differential protection of normal and malignant tissues against the cytotoxic effects of mechlorethamine. *Cancer Ther. Repts.*, **63**, 971.

YUHAS, J. M. (1979*b*). Misonidazole enhancement of acute and late radiation injury to the rat spinal cord. *Br. J. Cancer*, **40**, 161.

YUHAS, J. M. (1980*a*) On the potential application of radioprotective drugs in radiotherapy. In *Radiation–Drug Interactions in Cancer Management*, Ed. Sokol. New York: Wiley & Sons. (In press.)

YUHAS, J. M. (1980*b*) Active *versus* passive absorption kinetics as the basis for selective protection of normal tissues against alkylating agents by WR-2721. *Cancer Res.* (In press.)

YUHAS, J. M. & CULO, F. (1980) Selective inhibition of the nephrotoxicity of Cis-Platinum without altering its antitumor properties. *Cancer Ther. Repts.* (In press.)

YUHAS, J. M. & STORER, J. B. (1969) Differential chemoprotection of normal and malignant tissues. *J. Natl Cancer Inst.*, **42**, 331.


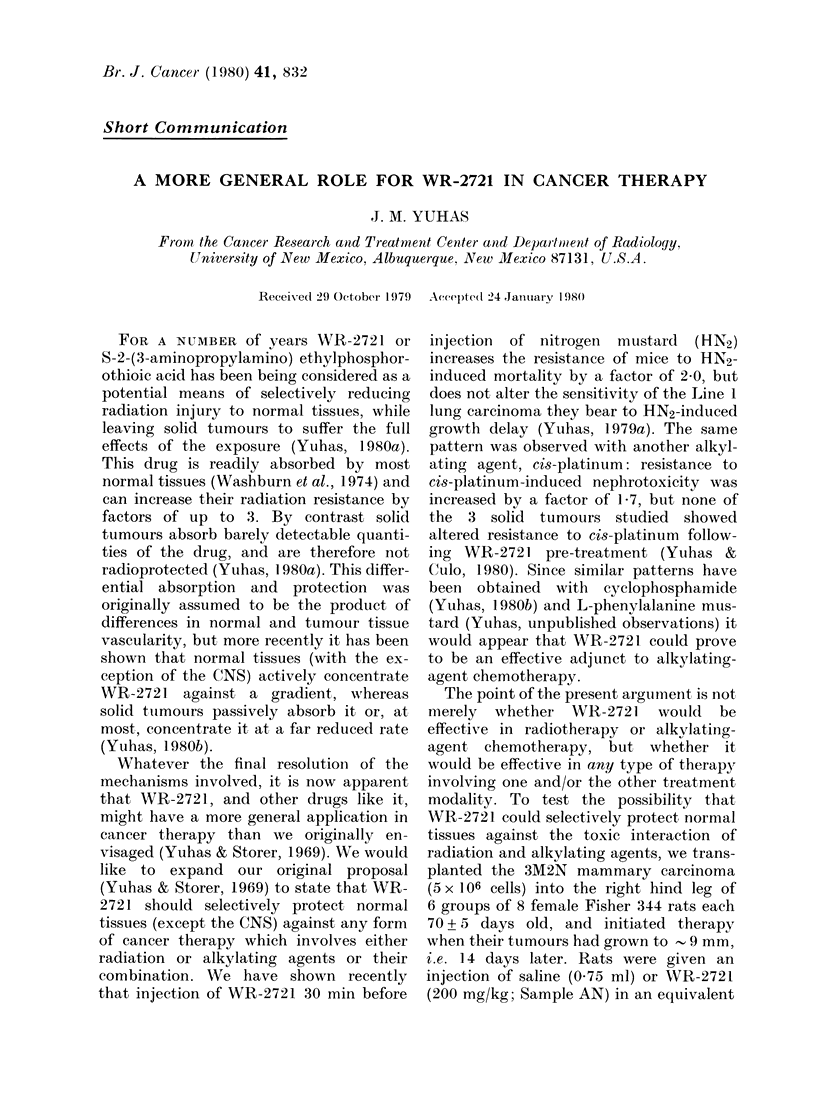

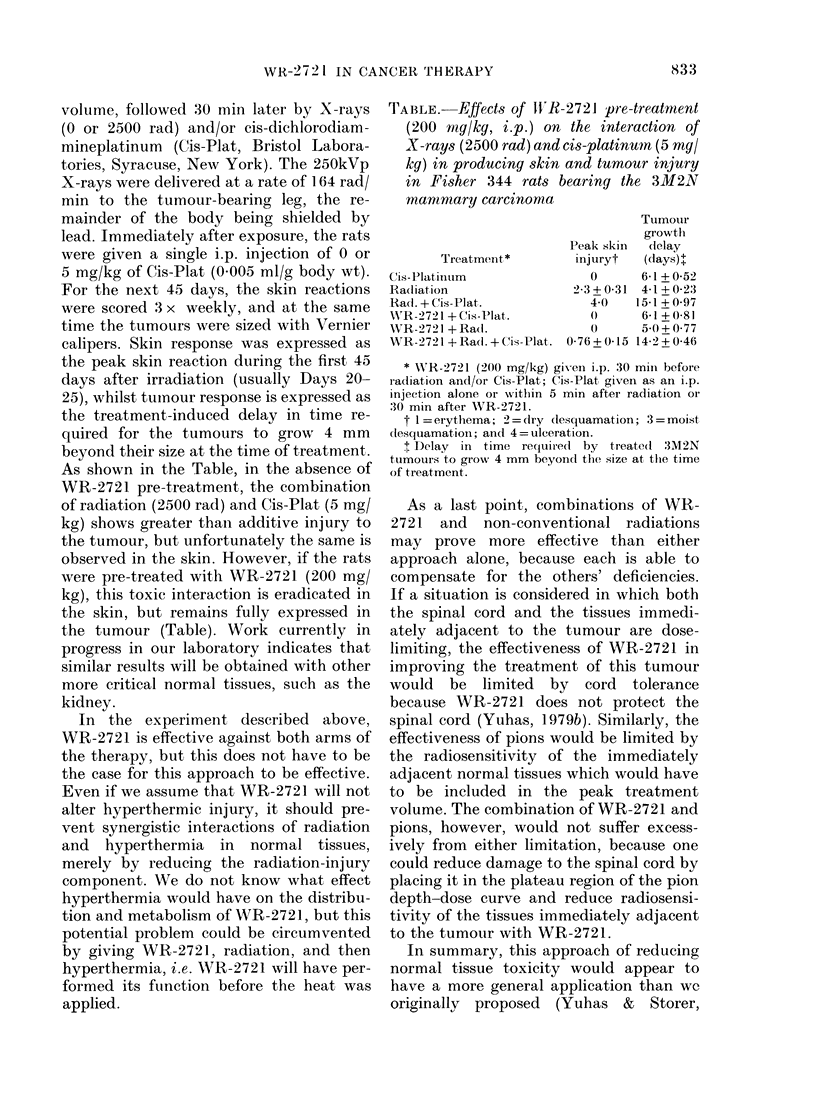

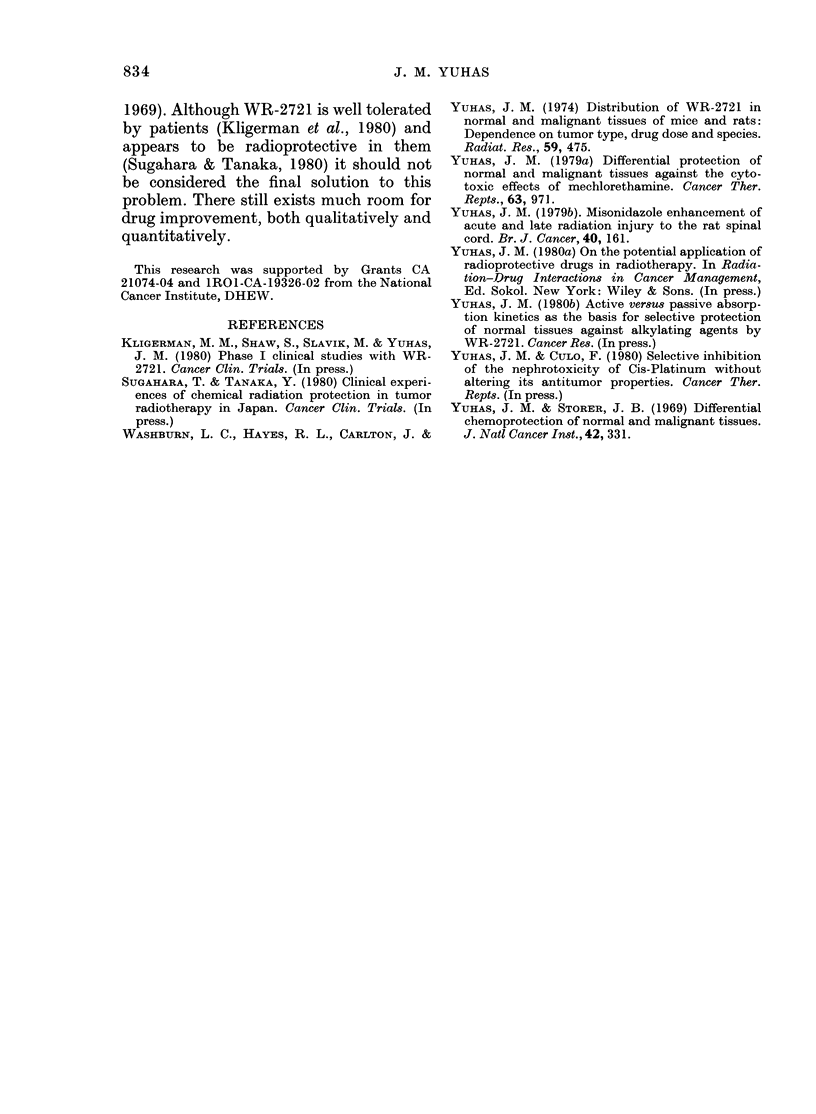


## References

[OCR_00259] Washburn L. C., Carlton J. E., Hayes R. L. (1974). Distribution of WR-2721 in normal and malignant tissues of mice and rats bearing solid tumors: dependence on tumor type, drug dose and species.. Radiat Res.

[OCR_00269] Yuhas J. M. (1979). Differential protection of normal and malignant tissues against the cytotoxic effects of mechlorethamine.. Cancer Treat Rep.

[OCR_00273] Yuhas J. M. (1979). Misonidazole enhancement of acute and late radiation injury to the rat spinal cord.. Br J Cancer.

[OCR_00295] Yuhas J. M., Storer J. B. (1969). Differential chemoprotection of normal and malignant tissues.. J Natl Cancer Inst.

